# A potential evolutionary trap for the extended phenotype of a nematomorph parasite

**DOI:** 10.1093/pnasnexus/pgae464

**Published:** 2024-10-15

**Authors:** Yuna Sawada, Nozomu Sato, Takeshi Osawa, Kazuma Matsumoto, Ming-Chung Chiu, Ryuichi Okada, Midori Sakura, Takuya Sato

**Affiliations:** Department of Zoology, Division of Biological Sciences, Graduate School of Science, Kyoto University, Sakyo-ku, Kyoto 606-8224, Japan; Graduate School of Urban Environmental Sciences, Tokyo Metropolitan University, Minami-Osawa 1-1, Hachiouji, Tokyo 192-0397, Japan; Graduate School of Urban Environmental Sciences, Tokyo Metropolitan University, Minami-Osawa 1-1, Hachiouji, Tokyo 192-0397, Japan; Retired researcher, Former institute: Forestry and Forest Products Research Institute, Tsukuba, Ibaraki 305-8687, Japan; Department of Entomology, National Taiwan University, No. 1, Sec. 4, Roosevelt Rd., Da’an District, Taipei City 106216, Taiwan; Department of Biology, Graduate School of Science, Kobe University, 1-1 Rokkodai-cho, Nada-ku, Kobe 657-8501, Japan; Department of Biology, Graduate School of Science, Kobe University, 1-1 Rokkodai-cho, Nada-ku, Kobe 657-8501, Japan; Center for Ecological Research, Kyoto University, 2-509-3 Hirano, Otsu, Shiga 520-2113, Japan

**Keywords:** behavioral manipulation, endosymbiont, hairworm, polarized light

## Abstract

Human activities introduce new environmental cues to wild organisms, leading to maladaptive behavioral and life history decisions known as the “evolutionary trap.” This trap is thought to be a major conservation concern for free-living organisms. However, it has never been studied in endosymbionts, one of the most successful and diverse life forms on Earth. Here, we examine this trap in the extended phenotype of a parasite that exploits the visual system of hosts to alter host behavior for its benefit. Arboreal mantids infected by nematomorph parasites are drawn to horizontally polarized light, thereby inducing them to enter the water. In this study, we found that the degree of linear polarization (DOP) of reflected light served as a reliable environmental cue for identifying perennial waters, where nematomorphs can survive in their aquatic life stage without drying out. Infected mantids exhibit attraction to horizontally polarized light with higher DOP in behavioral assays and jumped into pools reflecting light with higher DOP in field experiments. The asphalt road reflected horizontally polarized light closely resembling the polarization levels observed in perennial waters, likely leading to a higher prevalence of mantids on asphalt roads compared with those found in natural arboreal habitats. In a field experiment, we observed infected mantids walking on asphalt roads more often than on cement roads. These findings imply that evolutionary traps can endanger endosymbionts beyond their hosts that directly perceive environmental cues.

## Introduction

Organisms have evolved to use environmental cues, such as light, sound, and odor, to make various adaptive behavioral and life history decisions (1). Human activities have led organisms to rely on these formerly reliable cues for making maladaptive decisions, known as the “evolutionary trap” (1–3). This trap has been demonstrated in many free-living organisms (3–7) and is now recognized as a major threat to their biodiversity loss in human-dominated environments (1–3). However, the evolutionary trap has never been investigated in endosymbionts, even though they are the most successful and diverse life forms on earth, and their conservation is rapidly growing field (8, 9). In nature, various parasites manipulate host behavior as their extended phenotypes (10) to complete their life cycles (11). These parasites have evolved the ability to exploit their host's perception of environmental cues to induce behavioral changes in the host for their own benefit. For example, zombie ant fungi alter the visual perception of ants, causing them to climb trees and bite leaves, facilitating fungal spore transmission (12). Similar light-induced behavioral alterations have been observed in other manipulative parasites like baculovirus (13) and parasitic helminths (14, 15). Here, we hypothesized that human-induced environmental changes compel manipulated hosts to use formerly reliable environmental cues, leading to maladaptive behavior toward parasites, which should be regarded as an evolutionary trap for the extended phenotypes of endosymbionts.

**Fig. 1.**
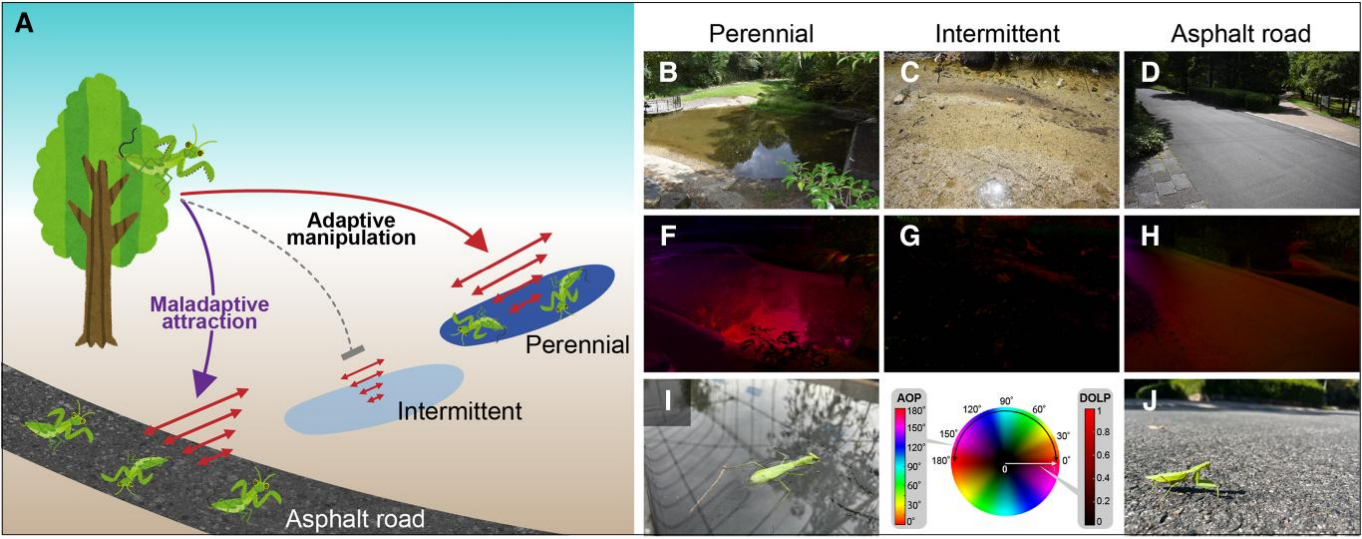
A potential evolutionary trap for the extended phenotype of a nematomorph parasite and related photos. A) Conceptual diagram of the hypothesis for the evolutionary trap. B–D) Color photos and (E–G) polarization images are shown for the representative perennial water body (B, E), intermittent water body (C, F), and asphalt road (D, G). In a color chart (bottom middle) synthesized using the angle of polarization (AOP, left bar) and DOP (right bar), the color indicates the AOP direction, and brightness indicates the DOP. The polarization images were taken at the Brewster’s angle (∼38° from the horizontal). H) A photo of a mantid jumping into the water. I) A photo of a mantid walking on the asphalt road.

Nematomorph parasites induce terrestrial hosts to enter the water, necessary for them to reproduce in aquatic habitats (16). Previously, we demonstrated that arboreal mantids (*Hierodula patellifera*) infected by nematomorph parasites (*Chordodes formosanus*) are attracted to horizontally polarized light, which is strongly reflected from water surfaces (17). This enhanced positive polarotaxis explains the water entry behavior of infected mantids (18) (Fig. 1A). Perennial water bodies (deeper with darker surfaces) are expected to reflect light with a higher degree of linear polarization (DOP; Fig. 1A, B, and F), whereas intermittent water bodies (shallower depths with brighter surfaces) likely reflect light with a lower DOP (Fig. 1A, C, and G) (19). Adult nematomorphs reproduce, and their larvae need to infect aquatic insect larvae in water without drying out until they are transmitted to terrestrial hosts upon an emergence of the aquatic insect hosts (16). Hence, nematomorphs would gain from inducing infected mantids to jump into perennial waters by attracting them toward light with a higher DOP (Fig. 1A).

However, this adaptive behavioral manipulation might prove maladaptive for nematomorphs in human-dominated environments. Particularly, nematomorph-infected mantids were often found on asphalt roads and were subsequently road-killed with the parasites (Fig. 1A). Asphalt roads share physical traits, such as smooth darker surfaces, with dark water surfaces and the ability to strongly polarize light, leading to polarized light pollution in many free-living organisms (5). This highlights the potential for asphalt roads introduced into nematomorphic habitats to mislead infected mantids. Herein, we hypothesized that the enhanced positive polarotaxis of infected mantids is adaptive for nematomorphs to selectively enter perennial water bodies but poses an evolutionary trap for the parasite if the asphalt roads run close to their habitats (Fig. 1A).

## Results and discussion

**Fig. 2.**
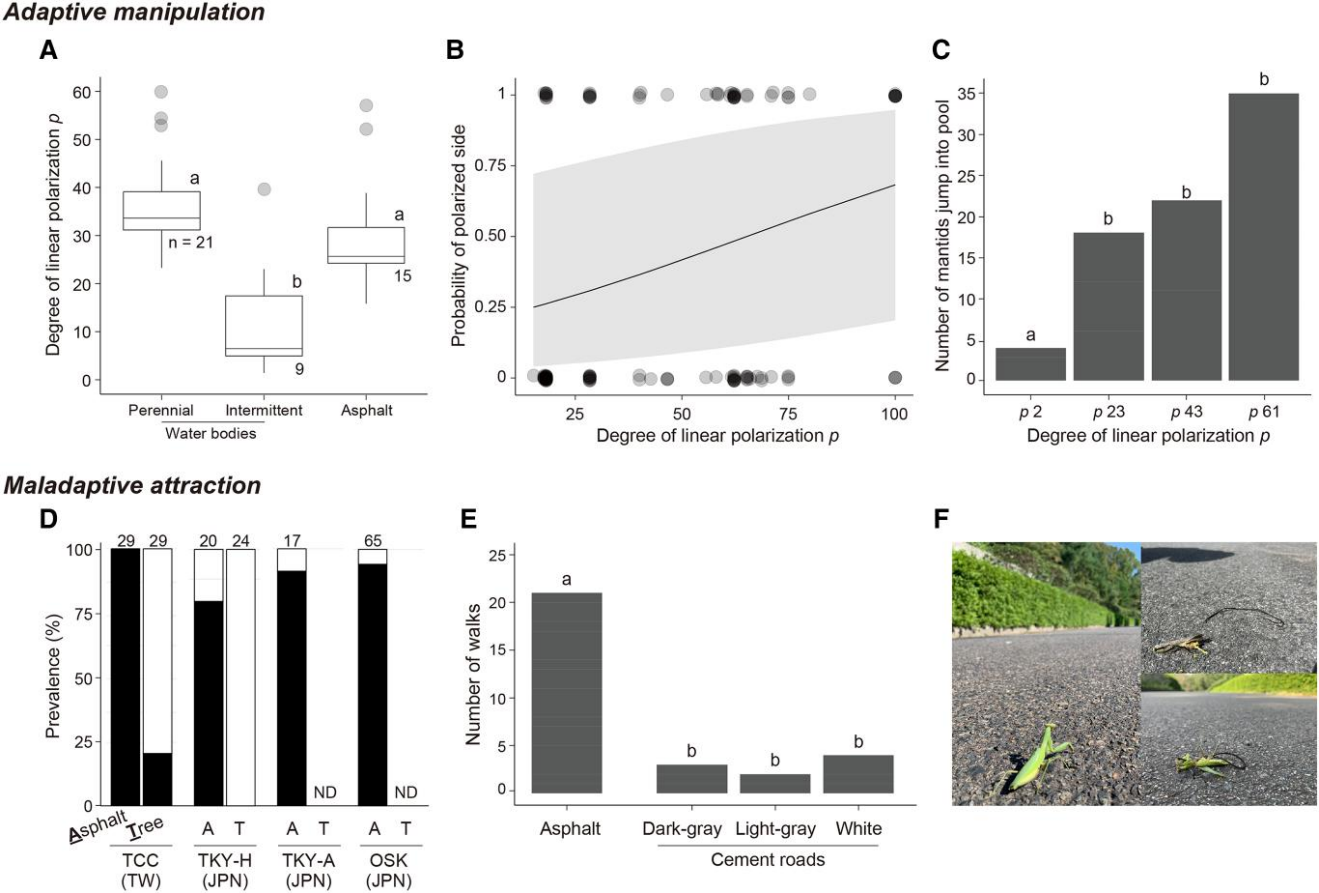
Adaptive manipulation to induce nematomorph-infected mantids to enter the perennial water bodies (A–C) and maladaptive attraction of these mantids to asphalt roads (D–F). A) The box plots of the DOP reflected from the water surfaces of perennial waters and intermittent waters and from asphalt roads. Whiskers are expressed as 1.5 times the interquartile range. B) Fitted logistic regression (with 95% CI) of the probability of polarized third selection based on the DOP. Transparent points represent the raw data. C) The number of infected mantids that entered the pools with different DOP. D) The prevalence of nematomorph (*C. formosanus*) infections on mantids (*H. patellifera* in TKY-A and OSK; *T. formosana* in TCC; and *H. chinensis* in TKY-H) in asphalt roads and natural arboreal habitats in four different sites in Taiwan (TW) and Japan (JPN). Numbers on the bars represent the sample sizes of the mantids. ND, no data available for the category. E) The number of walking events of the infected mantids on roads with different DOP. F) Photos of infected mantids walking and/or road-killed on asphalt roads. In (A) and (E), different letters within graphs indicate statistically significant differences.

We began to test these hypotheses by measuring the DOP (*d*) and relative intensity of light (RIL) reflected from various water bodies (*n* = 30) in nematomorph habitats in Japan. DOP was higher in perennial waters (mean ± SD: *d* = 38.1 ± 10.2%) than in intermittent waters (*d* = 12.7 ± 11.5%; Fig. 2A, Table S1), whereas RIL was largely overlapped between the 2 water types (RIL: 0.36 ± 0.33 in perennial waters and 0.22 ± 0.23 in intermittent waters; Table S1). As revealed using the logistic regression model, DOP was a reliable environmental cue to distinguish perennial waters from intermittent waters [generalized linear model with a binomial error distribution (GLM_binomial_): *z* = 2.54, *P* = 0.011]; no significant effects of RIL or its interaction with DOP were observed (Table S2). Subsequently, we tested whether the infected mantids (*H. patellifera*) were attracted to light with a higher DOP using a two-choice test, in which horizontally polarized light with different DOP (*d* = 15–100%) was illuminated (Fig. S1). Infected mantids were more likely to be attracted to horizontally polarized light with higher DOP [Fig. 2B; generalized linear mixed model with a binomial error distribution (GLMM_binomial_): *z* = 3.0, *P* = 0.003; Table S3], suggesting their sensitivity to changes in DOP. We further investigated whether the infected mantids selectively enter perennial waters that strongly reflect horizontally polarized light in a natural setting. Particularly, we conducted a field experiment, where four pools (W × H = 1.7 × 0.8 m with 0.05–0.48 m in depth) that reflected horizontally polarized light with different DOPs (mean *d* = 2, 23, 43, and 61%) were randomly placed in a greenhouse (L × W = 20 × 5 m; Fig. S2). Infected mantids entered pools with a higher DOP (*d* > 23%) more frequently than that with a low DOP (*P* = 2; Fig. 2C, Table S4). The value above which the probability of perenniality of natural water bodies was 50% or higher in the above GLM was *d* = 23%. We conclude that enhanced positive polarotaxis is a key factor not only in detecting water bodies (18) but also in directing infected mantids into perennial waters, where nematomorphs can survive in their aquatic life stage.

Next, we compared the DOP of light reflected from asphalt roads and water bodies to test the evolutionary trap for the nematomorphs. The DOP of the asphalt roads measured in the nematomorph habitats (mean ± SD: *d* = 30.2 ± 10.9%, range 15.8–57.0%) largely overlapped with that of the perennial water bodies (Fig. 2A, Table S1). In field observations in Taiwan and Japan, the prevalence of nematomorphs (*C. formosanus*) in mantids (*Titanodula formosana* in Taiwan; *H. chinensis* in Japan) collected from asphalt roads (>80%) was much higher than those found in their natural arboreal habitats (<20%; GLM_binomial_: *z* = 6.6, *P* < 0.001; Fig. 2D, Table S5). Observations of mantids (*H. patellifera*) at two other distant sites in Japan further supported the high nematomorph prevalence on asphalt roads (>92%; Fig. 1D), although we were unable to measure their prevalence in the natural habitats at these two sites. To further test the misleading of the infected mantids to the asphalt roads, we conducted a field experiment in which four different mimetic roads (one asphalt road and three cement roads with different colors; W × H = 1.8 × 0.9 m) that reflected horizontally polarized light with different DOP (asphalt roads: *d* = 30.7%; cement roads: *d* = 2.8–18.57%) were randomly placed in a greenhouse (L × W = 20 × 5 m; Fig. S3). The infected mantids walked more frequently on asphalt roads than on any other cement roads (Fig. 2E, Table S6). These results strongly suggest that the infected mantids experienced challenges in distinguishing asphalt roads from perennial water bodies with similarly high DOP and were attracted to asphalt roads. Dead nematomorphs were frequently observed [e.g. >100 worms in ∼500 m road in a sampling size in Osaka (OSK) each in 2021 and 2022], with infected mantids, which were exhausted and/or killed on asphalt roads (Fig. 2F), indicating substantial negative selection pressure on the nematomorphs' extended phenotype. However, its effect on population decline or evolutionary responses to these artificial environments remains uncertain.

This study presents lines of evidence to suggest that an evolutionary trap can affect organisms beyond those directly sensing environmental cues, that is, the evolutionary trap for the extended phenotype of parasites. This type of the evolutionary trap has never been reported before, except in this study. However, ∼40% of known species are parasitic (20), and phylogenetically diverse parasites, such as viruses, fungi, protozoa, and metazoans, have evolved strategies to manipulate hosts (11) with diverse sensory systems (1). Recent advancements in molecular biology techniques applied to nonmodel organisms have unveiled widespread host manipulations by parasites. These studies have begun to elucidate the neural and molecular mechanisms, often involving visual perception (12–15, 18), through which parasites exploit host sensory systems to manifest their extended phenotypes. Meanwhile, the global proliferation of human activities has altered the distribution of environmental stimuli, including artificial lights (3) and chemical agents (1). Therefore, our findings may be one of the several examples highlighting threats to parasites and their evolutionary trajectories, an issue demanding recognition and resolution.

## Supplementary Material

pgae464_Supplementary_Data

## Data Availability

All data for this study are available in Dryad: https://datadryad.org/stash/share/n4vN8sr7znayvAP_qgzD-Mt0BfdacWWbyHj5xdKMIXQ (DOI: https://doi.org/10.5061/dryad.jh9w0vtkb).

## References

[1] Dominoni DM , et al 2020. Why conservation biology can benefit from sensory ecology. Nat Ecol Evol. 4:502–511. 32203474 10.1038/s41559-020-1135-4

[2] Schlaepfer MA , Runge MC, Sherman PW. 2002. Ecological and evolutionary traps. Trends Ecol Evol. 17:474–480.

[3] Swaddle JP , et al 2015. A framework to assess evolutionary responses to anthropogenic light and sound. Trends Ecol Evol. 30:550–560. 26169593 10.1016/j.tree.2015.06.009

[4] Buxton RT , et al 2017. Noise pollution is pervasive in US protected areas. Science. 356:531–533. 28473587 10.1126/science.aah4783

[5] Horváth G , Kriska G, Malik P, Robertson B. 2009. Polarized light pollution: a new kind of ecological photopollution. Front Ecol Env. 7:317–325.

[6] Kyba CC , et al 2017. Artificially lit surface of earth at night increasing in radiance and extent. Sci Adv. 3:e1701528. 29181445 10.1126/sciadv.1701528 PMC5699900

[7] Robertson BA , Rehage JS, Sih A. 2013. Ecological novelty and the emergence of evolutionary traps. Trends Ecol Evol. 28:552–560. 23756104 10.1016/j.tree.2013.04.004

[8] Carlson CJ , et al 2020. A global parasite conservation plan. Biol Conserv. 250:108596.

[9] Doña J , Johnson KP. 2020. Assessing symbiont extinction risk using cophylogenetic data. Biol Conserv. 250:108705.

[10] Dawkins R . The extended phenotype: the long reach of the gene. Oxford (UK): Oxford University Press, 1999.

[11] Hughes DP , Libersat F. 2019. Parasite manipulation of host behavior. Curr Biol. 29:R45–R47. 30668944 10.1016/j.cub.2018.12.001

[12] Andriolli FS , et al 2019. Do zombie ant fungi turn their hosts into light seekers? Behav Ecol. 30:609–616.

[13] Liu X , et al 2022. Baculoviruses hijack the visual perception of their caterpillar hosts to induce climbing behaviour thus promoting virus dispersal. Mol Ecol. 31:2752–2765. 35258140 10.1111/mec.16425

[14] Cornet S , Franceschi N, Bauer A, Rigaud T, Moret Y. 2009. Immune depression induced by acanthocephalan parasites in their intermediate crustacean host: consequences for the risk of super-infection and links with host behavioural manipulation. Int J Parasitol. 39:221–229. 18708062 10.1016/j.ijpara.2008.06.007

[15] Ponton F , et al 2011. Water-seeking behavior in worm-infected crickets and reversibility of parasitic manipulation. Behav Ecol. 22:392–400. 22476265 10.1093/beheco/arq215 PMC3071748

[16] Hanelt B , Thomas F, Schmidt-Rhaesa A. 2005. Biology of the phylum Nematomorpha. Adv Parasitol. 59:243–305. 16182867 10.1016/S0065-308X(05)59004-3

[17] Wehner R . 2001. Polarization vision–a uniform sensory capacity? J Exp Biol. 204:2589–2596. 11511675 10.1242/jeb.204.14.2589

[18] Obayashi N , et al 2021. Enhanced polarotaxis can explain water-entry behaviour of mantids infected with nematomorph parasites. Curr Biol. 31:R777–R778. 34157257 10.1016/j.cub.2021.05.001

[19] Konnen G . Polarized light in nature. Cambridge (USA): CUP Archive, 1985.

[20] Dobson A , Lafferty KD, Kuris AM, Hechinger RF, Jetz W. 2008. Homage to Linnaeus: how many parasites? How many hosts? Proc Natl Acad Sci U S A. 105(Suppl 1):11482–11489. 18695218 10.1073/pnas.0803232105 PMC2556407

