# Supplementary material for: A potential evolutionary trap for the extended phenotype of a nematomorph parasite
Source: PNAS Nexus. 2024 Oct 15;3(10):pgae464. doi: 10.1093/pnasnexus/pgae464 (PMC11518923; doi:10.1093/pnasnexus/pgae464)
Supplement: pgae464_Supplementary_Data [file pgae464_supplementary_data.docx]

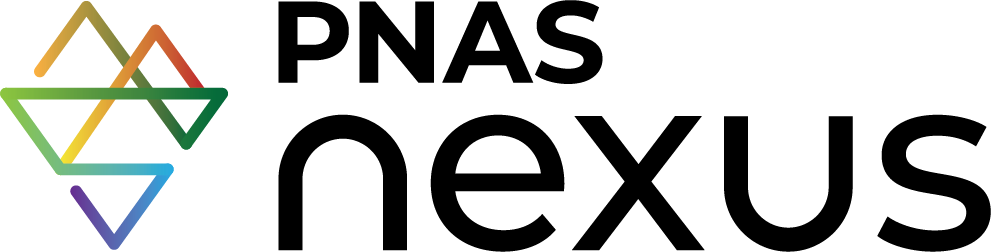


**Supplementary Information for**

**An evolutionary trap for the extended phenotype of a nematomorph parasite**

**Authors:** Yuna Sawada^1^, Nozomu Sato^2^, Takeshi Osawa^2^, Kazuma Matsumoto^3^†, Ming-Chung Chiu^4^, Ryuichi Okada^5^, Midori Sakura^5^, and Takuya Sato^6*^

**Affiliations:**

^1^ Department of Zoology, Division of Biological Sciences, Graduate School of Science, Kyoto University, Sakyo-ku, Kyoto, Japan 606-8224

^2^ Graduate School of Urban environmental Sciences, Tokyo Metropolitan University. Minami-Osawa 1-1, Hachiouji, Tokyo, Japan 192-0397

^3^ Retired researcher (Former institute: Forestry and Forest Products Research Institute, Tsukuba, Ibaraki, Japan 305-8687)

^4^ Department of Entomology, National Taiwan University. No. 1, Sec. 4, Roosevelt Rd., Da’an Dist., Taipei City, Taiwan 106216

^5^ Department of Biology, Graduate School of Science, Kobe University. 1-1 Rokkodai-cyo, Nada-ku, Kobe city, Japan 657-8501

^6^ Center for Ecological Research, Kyoto University. 2-509-3 Hirano, Otsu-city, Shiga, Japan 520-2113

*Corresponding author. Email: tsato@ecology.kyoto-u.ac.jp

†K. Matsumoto passed away on 31st March, 2024.

**This PDF file includes:**

Supplementary text (extended methods, including Figures S1-S3 and Tables S1-S8)

SI References

**Materials and Methods**

**Field collection of study animals**

Nematomorphs (Phylum: Nematomorpha) exhibit a complex life cycle, with two parasitic larval stages (aquatic and terrestrial) and a free-living adult stage (1). The final stage involves, the parasite manipulating its terrestrial insect host to enter streams or ponds (2), where the adult parasite must emerge and reproduce.

In this study, we used an arboreal mantid host belonging to the genus *Hierodula* (Mantodea: Mantidae), a common host of nematomorphs in the genus *Chordodes*, and is widely distributed from East to South Asia (3). Infected mantids often walk on asphalt roads surrounded by forests when they are manipulated by nematomorphs, leading to them being easily found and caught. We collected these presumably infected mantids (*Hierodula patellifera*) from three geographically distant locations in Osaka (OSK) and Tokyo (Akiruno: TKY-A and Hachioji: TKY-H) Japan. Presumably infected *Titanodula formosana* were collected in one location in Taiwan (Taichung city: TCC) (Table S7). Additionally, we collected mantids from their natural arboreal habitats at TKY-H and TCC. Furthermore, we collected *Hierodula chinensis*, which is an invasive species, in TKY-H, Japan. Invasive *H. chinensis* were infected with *C. formosanus*. To compare the prevalence of *C. formosanus* infections between asphalt roads and natural habitats we used the specimens from *H. chinensis*-*C. formosanus* association (see below). We identified the nematomorph specimens used in this study using the mitochondrial COI gene(4) and confirmed that all belonged to one species, *Chordodes formosanus* (Table S7).

**Laboratory maintenance and criteria**

Mantids were individually kept in plastic containers, with food provided *ad libitum* (approximately 3 house crickets per 2–3 d), humidified by an atomiser, and at 25°C under a 12:12-h light:dark cycle.

The mantids were considered to be manipulated when the head of a nematomorph (“worm”) was visible at a mantid’s posterior end, for three reasons. Firstly, adult worms form an open wound on the posterior end of the host from which they poke their heads out to assess the external environment before emerging from their hosts (5). Secondly, only worms at this stage emerged from the hosts when we dunked the hosts’ abdomens in water(6). Thirdly, when worms (*Chordodes fukuii*) were at this stage, mantid hosts (*Tenodera angustipennis*) were previously found to increase their activity levels and exhibited unique gene expression patterns (6). We continued to assess the posterior ends of the hosts daily until worm heads or host deaths were observed.

The mantids presumed to be manipulated were used for behavioural experiments as manipulated hosts within a week before they weakened. All experiments were conducted from late September (16 October 2021; 18 September 2022; and 6 September 2023) to early November (4 November 2021; 4 November 2022; and 27 October 2023), when they were manipulated by natural nematomorphs.

**Characteristics of the reflected lights**

We measured the degree of linear polarisation (DOP), light intensity, and light spectrum reflected from various water bodies (n = 30) and asphalt roads (n = 15) in the habitats of nematomorphs to test whether the DOP is an important environmental cue for distinguishing perennial or intermittent water bodies from asphalt roads. These light characteristics were measured in 2021-2023 under clear skies between 10 am and 3 pm from late September to early November, the time of day and period when nematomorphs manipulated mantid hosts (7).

**DOP**: DOP is a quantity used to describe the proportion of polarised electromagnetic waves. It is usually expressed as *d*, ranging from 0% to 100%, that is, perfectly polarised and unpolarised waves have *d* = 100% and *d* = 0%, respectively. Herein, according to a video polarimetry method described by Horváth *et al.* (8), we calculated the DOP as brightness. We followed the first three steps of the video polarimetry, that is, photographing (described as video recording in the original method (8)) in a field, digitisation and computation in a laboratory. A polarisation camera (VCXU-50MP Baumer, Inc. Switzerland) was set to the sunlight at a horizontal position of 22–64 cm in height. We confirmed that the height of the camera did not affect the DOP calculation as long as we measured them at the same angle. The camera angle was fixed at the Brewster’s angle, which is the angle of incidence at which light reflected from a surface is perfectly polarised using a tripod. A common linearly polarising filter (LA-105, Nippon Medical & Chemical, Inc. Japan) was mounted in front of the camera’s objective lens and three images under the “Intensity Mode” of the camera were taken with three different polariser alignments (φ = 0º, 45º and 90º) by manually rotating the filter by 45º. In the laboratory, we obtained a mean brightness from the central squares of 300 × 300, 400 × 400 and 500 × 500 pixels for each image taken with the three different alignments and fitted their modulation to a sinusoid (*I* = *A* sinφ + *B*). Subsequently, we calculated the DOP (*d*) using the following equation: *p* = (*I_max_*−*I_min_*)/(*I_max_*+*I_min_*), where *I_max_* and *I_min_* are maximum and minimum values, respectively, that can be extracted from the sinusoid. We confirmed that the mean brightness from the three different squares for each image substantially reduced the effect of the inevitable inclusion of small particles and/or shadows on the brightness values. The DOPs were calculated for the wavelength range of 400–900 nm, which is the default setting in the polarisation camera that we used.

**Relative intensity of light (RIL)**: Previous reports have argued that the attraction of the response to light intensity is a factor that induces nematomorph hosts to find and enter water bodies (9). However, we expected that light intensity would not be an environmental cue for finding perennial water bodies (7) because the reflected light intensity would be even higher in intermittent waters than in perennial waters. We tested this by measuring the light intensity of direct sunlight and reflected light from water surfaces and asphalt roads using a light analyser (LA-105 NIPPON MEDICAL & CHEMICAL, Inc. Japan). The measurements were taken at the same locations, heights and angles as those used for the imaging polarimetry. The intensity of sunlight, which varied considerably from minute to minute, even on sunny days, strongly influenced the absolute values of the reflected light intensity. Thus, the relative intensity of the reflected light (RIL) was calculated by dividing the intensity of the reflected light by that of sunlight. The RIL was calculated for the wavelength range of 380–780 nm, a default setting in the light analyser.

**Perenniality of water bodies:** All water bodies (streams, ponds and ditches) in the nematomorph habitats were observed at least three times between September 2022 and November 2023 to determine whether they had dried. Water bodies were subsequently classified as “perennial” only if the water did not dry up during this period.

**Statistical analysis:** A generalised linear model (GLM; MASS function in the R package ‘stats ’) with a binomial distribution and logit link function was used to test whether the perenniality of water bodies was associated with DOP, RIL and their interactions. The fixed and interaction terms were excluded from the final model if they were not significant in the GLM model.

GLM (gaussian distribution and identity link function) followed by Tukey’s honestly significant difference test was performed to test the differences in DOP among perennial waters, intermittent waters and asphalt roads in the nematomorph habitats.

**Two-choice test in the laboratory**

We conducted a two-choice test in a laboratory from late September to early November from 2021 to 2023, when the nematomorphs manipulated their mantid hosts in natural habitats to test whether the infected mantids were attracted to light with a higher DOP. The light choice box (W × L× H: 0.3 × 0.9 × 0.3 m) has two windows (5 cm × 5 cm), one at each end, to provide polarized and unpolarized light stimuli of the same light intensity and spectrum (Figure S1). The box has a vertical tube (5 cm ø, 7.5 cm long) at the centre of the base, through which a mantid was guided to the arena. Additionally, it has a removable cover with a circular hole (5 cm ø) at the centre of the top through which light is shone to guide the mantid into the box. Horizontally polarised light stimuli with different DOPs were produced using a depolarising matte tracing paper (No. 396863; Ostrich Co., Ltd. JAPAN), and a linearly polarising sheet (MLPH40LS-2; Me Can Imaging, Inc. JAPAN) in that order from the light source by inserting a series of 15 slightly depolarised overhead projector (OHP) sheets between a polarising sheet and a window of the choice box. This enabled us to produce DOPs (*d*) that ranged from 15.3% (15 OHP sheets) to 79.9% (one sheet). The DOP at a given setting was calculated three times using the same protocol described above, and its consistency was confirmed. The unpolarized light stimulus was produced by setting the two depolarising and polarising sheets in reverse order. We provided the same intensity of light stimuli from both sides of the light windows, by adjusting the LED light source (white colour mode, SEMLOS clip light; SEMLOS JAPAN). A luminance meter (TMS870; Ichinen Tasco Co., Ltd. JAPAN) was used to adjust the light intensity from the two windows. We confirmed that the degree of polarisation was approximately 1.0 for polarised light stimuli and 0 for unpolarized light stimuli when no OHP sheets were inserted between them. Additionally, the inner surface of the choice box was covered with matte white paper (LIFELEX KH3141, KohnanShoji Inc. Japan) and the specular reflection and unwanted polarisation signals were carefully determined to be negligible on the inner surface. The light intensity was set at 6000 lx throughout the experiment, which roughly corresponded to the intensities of the water surface reflections of light during cloudy middays. In our previous study, this light intensity was strong enough to induce positive polarotaxis in infected mantids(7). We randomly changed the polarised and unpolarized sides in a given choice box to avoid unexpected biases during the experiment. Four choice boxes of the same design were developed and used to simultaneously test the four mantids.

We previously found that even presumably manipulated mantids were not always attracted to horizontally polarised light stimulus, potentially due to their time-specific manipulation (7) or counter adaptation of hosts to the parasite manipulation (10). We explicitly tested the effect of DOPs on positive polarotaxis by conducting a screening test to ensure that the infected mantids used in the experiment were attracted to horizontally polarised light, at least at the beginning of the behavioural assay. Specifically, the mantids were individually placed in one of the other three choice boxes for approximately 1 h, from 10 am to 3 pm. Subsequently, we observed the behaviour of the mantids approximately every 15 min and only used individuals in the experiment if they were attracted to perfectly horizontally polarised light (*d* = 100%). Consequently, 51 of the 239 infected mantids were used in the choice test.

We placed an infected mantid at the entrance of a vertical tube (Figure S1) and guided it to the arena by illuminating it above in each trial. At the end of 10 min, the mantid location was recorded as the polarised, middle or unpolarized third of the box (7). Each mantid was assessed once in each DOP treatment but was randomly assigned to treatments with different DOPs (mean ± SD = 2.8 ± 0.7 times, range: 2–4 times). The mantids were kept in a dark environment for at least 15 min between trials to reduce the potential influence of the preceding trial.

**Statistical analysis:** A generalised linear mixed model (GLMM; glmer function in R package lme4) with a binomial distribution and logit link function was used to test whether the presence (1) or absence (0) of a mantid in the polarised third was associated with DOPs. Individual mantids (i.e. repeated measures at different DOPs), the year of the experiment and the ID of the choice boxes were included as the random effects.

**Field experiment for water-entry of mantids**

We conducted a pool choice experiment to test whether infected mantids were more likely to enter pools that reflected horizontally polarised light with higher DOPs (i.e. perennial waters). The DOP increases with increasing depth and darkness of the water bottom (11). Therefore, we created four pools that reflected horizontally polarised light with different DOPs by varying the depth and darkness of the bottom (Figure S2). We confirmed that the relative light intensity was largely overlapped among the four pools with no significant difference among them (ANOVA: *F_3, 12_*＝0.38, *P* = 0.77 in 2022; *F_3, 8_*＝2.08, *P* = 0.18 in 2023). Additionally, the spectral components of the reflected light were similar among the four pools, especially in the wavelength range of 400–600 nm where the spectral sensitivity of the mantids was high (12) (Figure S2). The four pools were placed 2.0 m apart in a mesh house (W × L× H: 5.4 × 20 × 2.5 m) made of 0.98-mm nylon mesh net supported on aluminium frames. The arboreal mantids were randomly released in potted oak (*Quercus myrsinifolia*) and tropical ash (*Fraxinus griffithii*) or in the mesh net. The four pools lay in the north and south directions, without any buildings or trees preventing insolation. Thus, the four pools would reflect direct sunlight with similar diurnal patterns. We randomly rotated the four pools approximately every week each year to avoid the effect of location on the frequency of water entry by the infected mantids.

The experiment was conducted from late September to late October of 2022 and 2023 on an experimental farm at the Center for Ecological Research, Kyoto University (34°97′N, 135°96′E). This experiment coincided with the season in which *C. formosanus* manipulates their mantid hosts in this region (7). During the experiment, 286 infected mantids were released into the mesh house, with 11 ± 7 mantids (range: 1-27 mantids) released at intervals of 3 ± 2 d (range: 1–10 days). The mantids were individually marked on their forewings with nail varnish to allow tracking. We quantified the time (hours in the day) and location (one of the four pools) of the mantid entry into the water using time-lapse cameras set above each pool; each camera captured hourly pictures throughout the experiment.

**Statistical analysis:** We used log-linear models for contingency tables (GLM with a Poisson distribution and a log-link function) to test whether the proportional compositions of the mantids jumping into the four pools were associated with DOP and the location of the pools (outside vs. inside). We tested the effect of location because the infected mantids were frequently observed at the northern and southern ends of the mesh house. In the GLM analysis, the fixed and interaction terms were excluded from the final model if they were insignificant (*P* > 0.05). We conducted post hoc log-likelihood ratio tests with Bonferroni correction (*P* < 0.0083 considered to be statistically significant) when the fixed effect was significant. Table S8-1 is a contingency table summarising the number of mantids observed in each pool and location.

**Prevalence in asphalt and natural habitat**

A GLM (MASS function in R) with a binomial distribution and logit link function was performed to test whether the infection probability [infected (1) or uninfected (0)] was associated with habitat (natural vs. asphalt road), location (TKY-H vs. TCC) and their interaction. In the GLM analysis, the fixed and interaction terms were excluded from the final model if they were not significant.

**Field experiment for mantids’ walking**

We conducted a road-choice experiment to test whether the infected mantids were more likely to walk on asphalt roads, which strongly reflect horizontally polarised light, than on cement roads, which weakly reflect it. The mimetic asphalt roads (DOP, approximately *d* = 30.7%) and three other mimetic cement roads (dark-grey: *d* = 18.6%, light-grey: *d* = 2.8%, white: *d* = 7.3%) were placed 2.0 m apart in the same mesh house used in the field experiment described above (Figure S3). We confirmed that the relative light intensity overlapped among the four roads (asphalt = 0.51–0.78, dark grey = 0.46–0.51, light grey = 0.60–0.65 and white = 0.70–0.97). The spectral components of the reflected light were similar among the four roads, particularly in the wavelength range of 400–600 nm (Figure S2). The four roads lay in the north and south directions. Therefore, they would reflect direct sunlight in a similar diurnal pattern. The four roads were randomly rotated approximately every five days (three times during the experiment) to avoid the effect of location on the walking frequency of the infected mantids.

The experiment was conducted from 22 October to 11 November 2022. During the experiment, 22 infected mantids were released into the mesh house, with 1-10 mantids released at intervals of 1–6 d. The mantids were individually marked on their forewings with nail varnish to allow tracking. We quantified the number of walking events on each road using time-lapse cameras set above each road. Each camera captured an image every minute throughout the experiment. We counted mantid walking events according to the following four criteria to minimise the overestimation of the attractiveness of the asphalt road. First, a mantid walking on a given road at a given time of the day was counted as one walking event, regardless of the duration of its stay. Second, if a given mantid walked on the same road at different times of the day, these were considered to be one walking event. Third, if a mantid walked on different roads on the same day, these were counted as different walking events. Finally, if a mantid walked on the same road on different days, they were counted as separate walking events.

**Statistical analysis:** We used log-linear models for a contingency table (GLM with a Poisson distribution and log-link function) to test whether the proportional compositions of the mantids that walked on the four roads were associated with the types and locations of the roads (outside vs. inside). In the GLM analysis, the fixed and interaction terms were excluded from the final model if they were insignificant (*P* > 0.05). We conducted post hoc log-likelihood ratio tests with Bonferroni correction (*P* < 0.0083 considered to be statistically significant) when the fixed effect was significant. Table S8-2 is a contingency table summarising the number of mantids observed in each pool and location.

**Acknowledgments**

We thank A. Imagawa, D. Iijima, K. Ushirokawa and T. Yamashita for sample collection. We acknowledge A. Matsumoto and S. Yoshinami for their substantial assistance on our field experiment.

**References**

[dataset] Y. Sawada, N. Sato, T. Osawa, K. Matsumoto, M-C. Chiu, R. Okada, M. Sakura, T. Sato (2024) Polarized light characteristics, biological information on mantids and observational/ experimental data. In Dryad [*https://datadryad.org/stash/share/n4vN8sr7znayvAP_qgzD-Mt0BfdacWWbyHj5xdKMIXQ*](https://datadryad.org/stash/share/n4vN8sr7znayvAP_qgzD-Mt0BfdacWWbyHj5xdKMIXQ) (DOI: <https://doi.org/10.5061/dryad.jh9w0vtkb>)

1. B. Hanelt, F. Thomas, A. Schmidt-Rhaesa, Biology of the Phylum Nematomorpha. **59**, 243-305 (2005).

2. F. Thomas *et al.*, Do hairworms (Nematomorpha) manipulate the water seeking behaviour of their terrestrial hosts? *J. Evol. Biol.* **15**, 356-361 (2002).

3. M.-C. Chiu, C.-G. Huang, W.-J. Wu, S.-F. Shiao, A new horsehair worm, Chordodes formosanus sp. n.(Nematomorpha, Gordiida) from Hierodula mantids of Taiwan and Japan with redescription of a closely related species, Chordodes japonensis. *ZooKeys*, 1 (2011).

4. T. Sato, K. Watanabe, S. Tamotsu, A. Ichikawa, A. Schmidt-Rhaesa, Diversity of nematomorph and cohabiting nematode parasites in riparian ecosystems around the Kii Peninsula, Japan. *Can. J. Zool.* **90**, 829-838 (2012).

5. M. G. Bolek, A. Schmidt-Rhaesa, L. C. De Villalobos, B. Hanelt, Phylum Nematomorpha. 10.1016/b978-0-12-385026-3.00015-2, 303-326 (2015).

6. T. Mishina *et al.*, Massive horizontal gene transfer and the evolution of nematomorph-driven behavioral manipulation of mantids. *Curr. Biol.* **33**, 4988-4994. e4985 (2023).

7. N. Obayashi *et al.*, Enhanced polarotaxis can explain water-entry behaviour of mantids infected with nematomorph parasites. *Curr. Biol.* **31**, R777-R778 (2021).

8. G. Horváth, D. Varjú, Polarization pattern of freshwater habitats recorded by video polarimetry in red, green and blue spectral ranges and its relevance for water detection by aquatic insects. *The Journal of experimental biology* **200**, 1155-1163 (1997).

9. F. Ponton *et al.*, Water-seeking behavior in worm-infected crickets and reversibility of parasitic manipulation. *Behav. Ecol.* **22**, 392-400 (2011).

10. R. Poulin, J. Brodeur, J. Moore, Parasite manipulation of host behaviour: should hosts always lose? *Oikos*, 479-484 (1994).

11. G. Konnen, *Polarized light in nature* (CUP Archive, 1985).

12. C. Sontag, Spectral sensitivity studies on the visual system of the praying mantis, Tenodera sinensis. *The Journal of General Physiology* **57**, 93-112 (1971).

**Figures S1-S3**


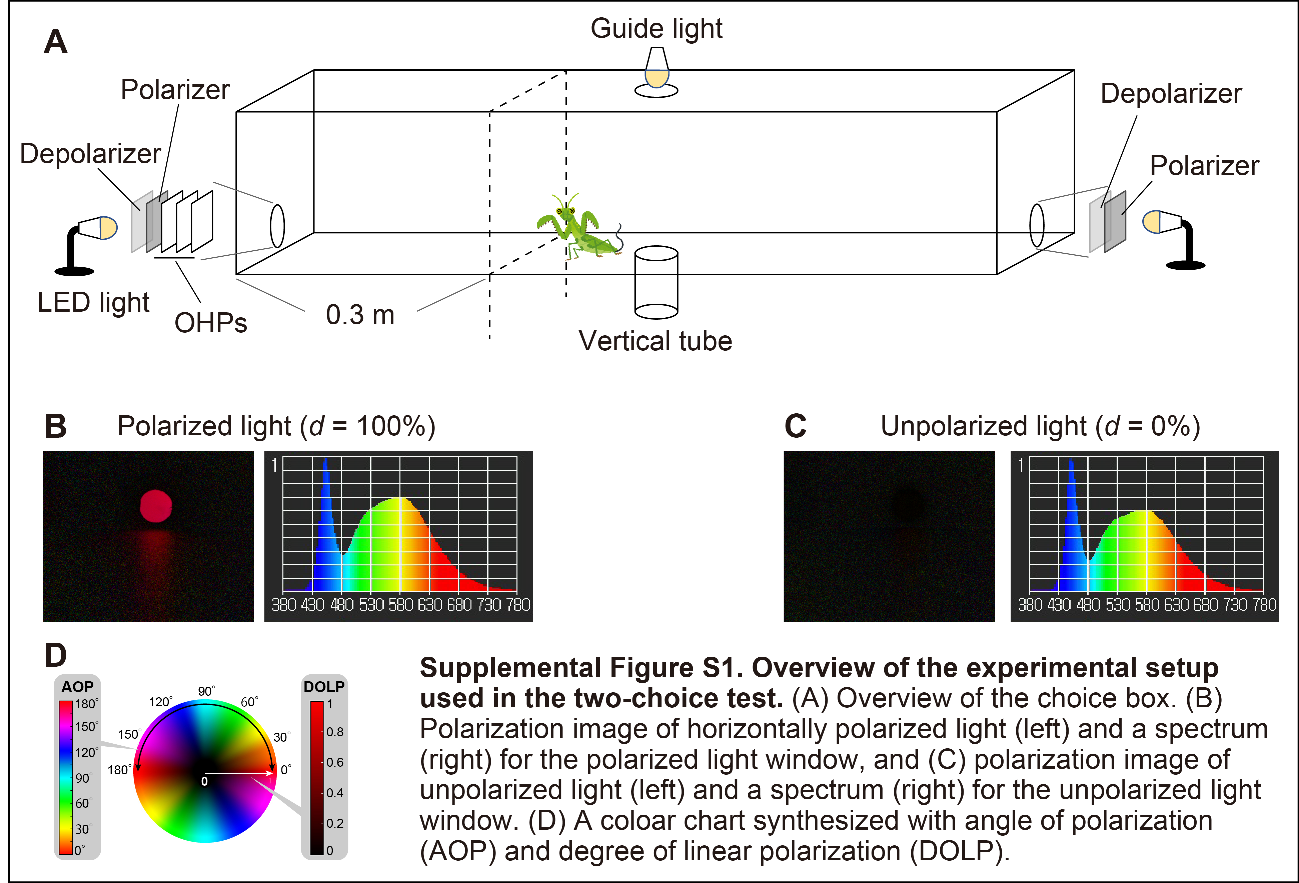


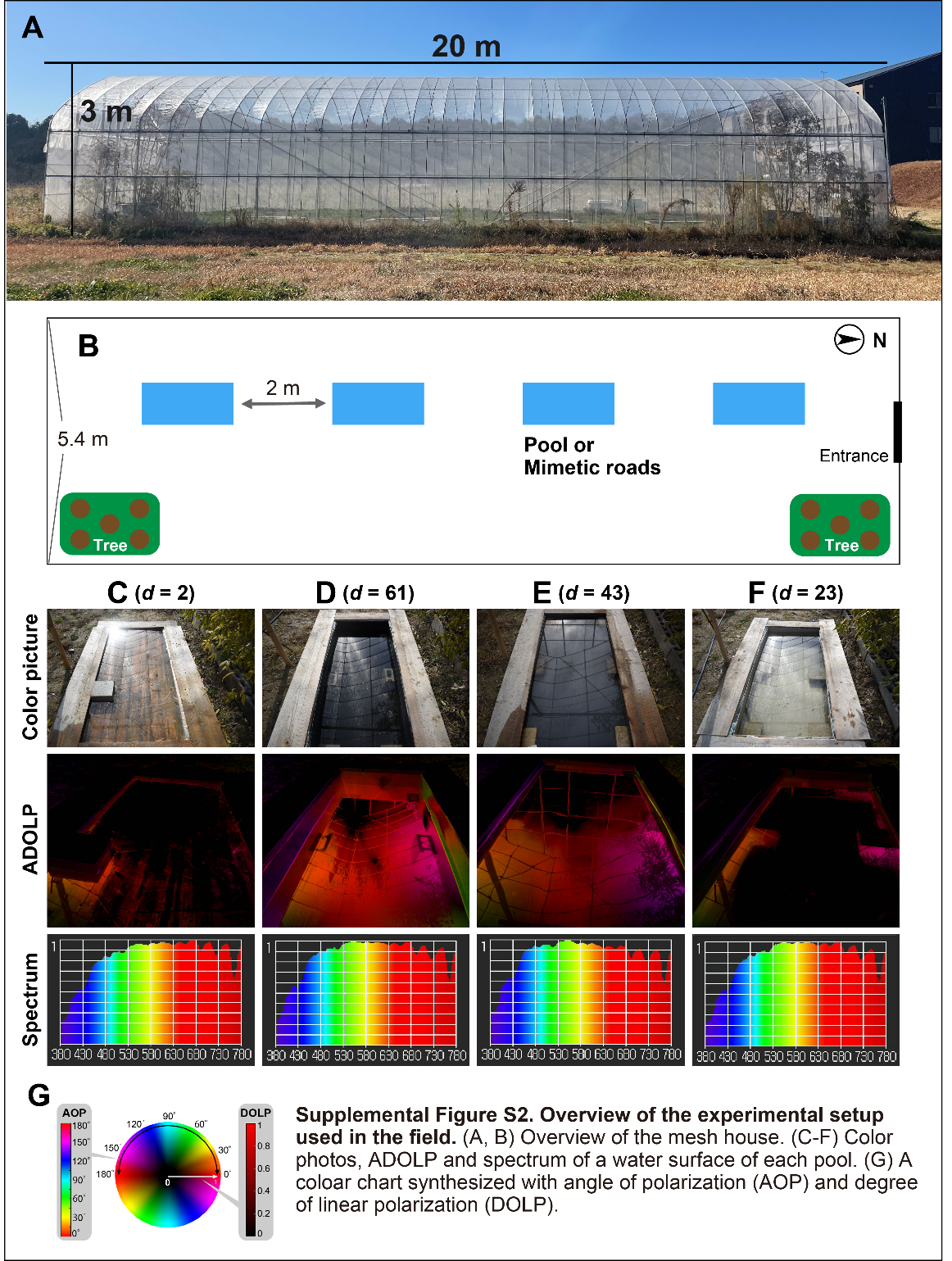


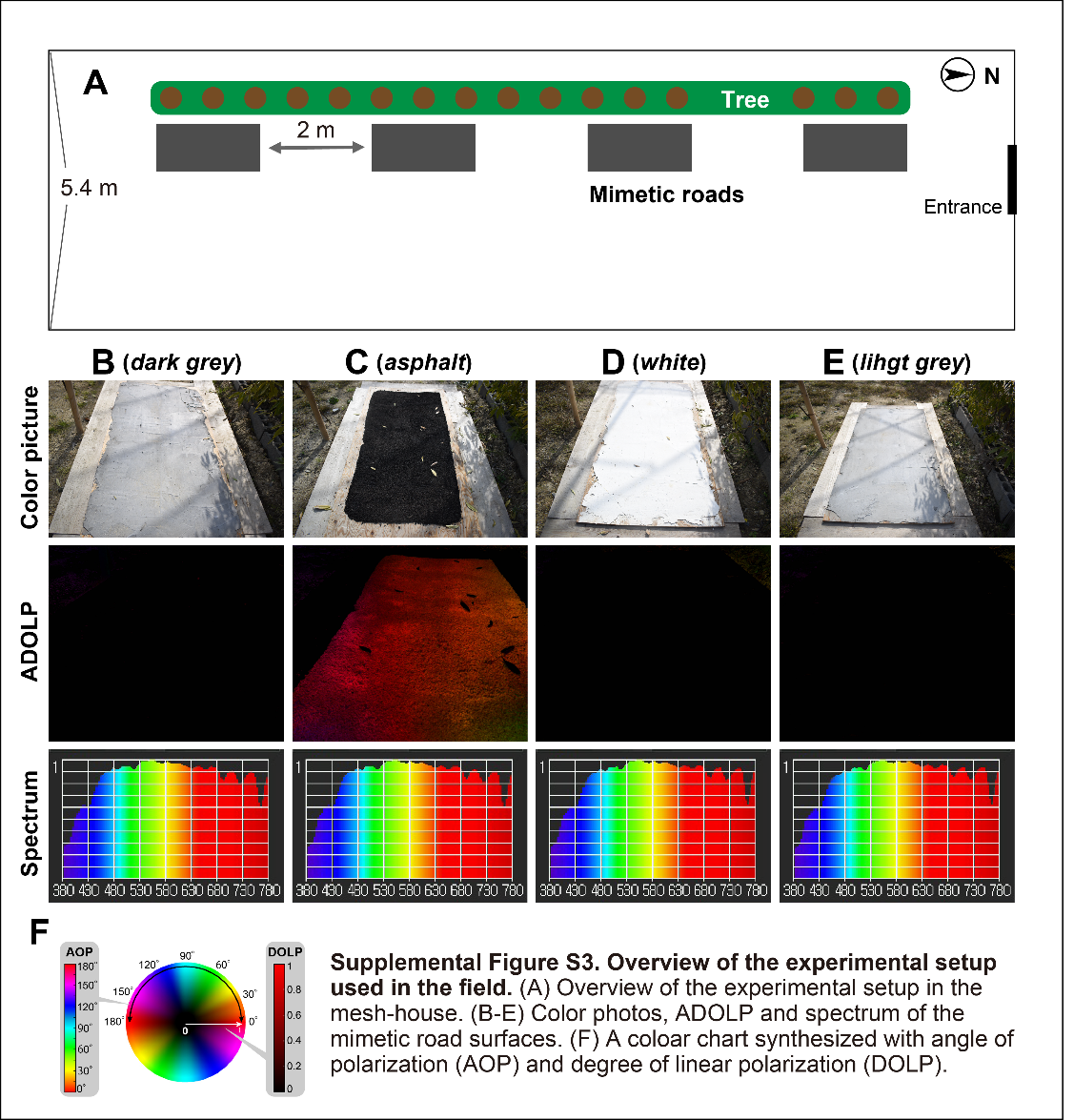


**Tables S1-S8**
